# Treatable Vascular Risk and Cognitive Performance in Persons Aged 35 Years or Older: Longitudinal Study of Six Years

**DOI:** 10.14283/jpad.2018.47

**Published:** 2018-12-14

**Authors:** Marlise E.A. van Eersel, H. Joosten, R.T. Gansevoort, J.P.J. Slaets, G.J. Izaks

**Affiliations:** 1 University of Groningen, University Medical Center Groningen, University Center for Geriatric Medicine, Internal Postcode: AA41, PO Box 30.001, 9700 RB, Groningen, The Netherlands; 2 Department of Internal Medicine, Maastricht University Medical Center, Maastricht, The Netherlands; 3 University of Groningen, University Medical Center Groningen, Department of Nephrology, Groningen, The Netherlands

**Keywords:** Cognitive performance, treatable vascular risk, longitudinal analysis, cardiovascular disease, preventing cognitive impairment

## Abstract

**Background:**

Poor cognitive performance is associated with high vascular risk. However, this association is only investigated in elderly. As neuropathological changes precede clinical symptoms of cognitive impairment by several decades, it is likely that cognitive performance is already associated with vascular risk at middle-age.

**Objectives:**

To investigate the association of cognitive performance with treatable vascular risk in middle-aged and old persons.

**Design:**

Longitudinal study with three measurements during follow-up period of 5.5 years.

**Setting:**

City of Groningen, the Netherlands.

**Participants:**

Cohort of 3,572 participants (age range, 35–82 years; mean age, 54 years; men, 52%).

**Exposure:**

Treatable vascular risk as defined by treatable components of the Framingham Risk Score for Cardiovascular Disease at the first measurement (diabetes mellitus, smoking, hypercholesterolemia and hypertension).

**Measurements:**

Change in cognitive performance during follow-up. Cognitive performance was measured with Ruff Figural Fluency Test (RFFT) and Visual Association Test (VAT), and calculated as the average of the standardized RFFT and VAT score per participant.

**Results:**

The mean (SD) cognitive performance changed from 0.00 (0.79) at the first measurement to 0.15 (0.83) at second measurement and to 0.39 (0.82) at the third measurement (Ptrend<0.001). This change was negatively associated with treatable vascular risk: the change in cognitive performance between two measurements decreased with 0.004 per one-point increment of treatable vascular risk (95%CI, -0.008 to 0.000; P=0.05) and with 0.006 per one-year increment of age (95%CI, -0.008 to -0.004; P<0.001).

**Conclusions:**

Change in cognitive performance was associated with treatable vascular risk in persons aged 35 years or older.

## Introduction

**S**everal studies have shown that poor cognitive performance is associated with vascular risk factors in persons aged 60 years or older (1). As neuropathological changes start several decades prior to the clinical expression of cognitive impairment (2), it is likely that cognitive performance is already associated with vascular risk factors at middle-age. However, vascular risk factors in middle-aged persons often are only marginally elevated if considered separately. Nevertheless they result in a clearly increased vascular risk if considered together (3, 4, 5, 6), which may contribute to the onset of neurodegenerative changes in the brain (7). Therefore, it is essential to know whether cognitive performance is associated with a high vascular risk in middle-aged persons.

Vascular risk is usually estimated with multicomponent risk scores that predict an individual's risk of a vascular event within the next years (3, 4, 5, 6). These vascular risk scores are largely based on age. However, although age is a major vascular risk factor, it is not amenable to treatment. For effective prevention of cognitive impairment it is essential to know whether cognitive performance is associated with treatable vascular risk based on treatable components like, for example, diabetes mellitus, hypertension and hypercholesterolemia. Up till now, three longitudinal studies have found a negative association of cognitive performance with treatable vascular risk independent of age (8, 9, 10). However, one study included a relatively small sample of 235 men aged 60 years or older (8), whereas the two other studies mainly included even older persons from the same source population (the Alzheimer's Disease Centers) (9, 10). Furthermore, in these three studies, the treatable vascular risk was based on a stroke-specific risk score and did not include the risk of cardiac or peripheral vascular events (4). Therefore, it is still unclear whether cognitive performance is associated with general treatable vascular risk, and not only with stroke-specific risk. Finally, the association between cognitive performance and treatable vascular risk is not yet investigated in middle-aged persons since current data are only available for elderly (8, 9, 10).

Therefore, this longitudinal study aims to investigate the association of cognitive performance with treatable (general) vascular risk independent of age over a followup period of six years in both middle-aged as old persons.

## Methods

### Study design

This study was part of the Prevention of REnal and Vascular ENd-stage Disease (PREVEND) cohort. The PREVEND study is a prospective cohort study investigating the natural course of microalbuminuria and its association with renal and cardiovascular disease. Details of the PREVEND study have been described elsewhere (11, 12). Briefly, at baseline 8,592 participants aged 28–75 years were selected from inhabitants of the city of Groningen (Netherlands) based on their urinary albumin excretion. These participants completed the baseline survey in 1997–1998 and were followed over time. Surveys included assessment of demographic and vascular risk factors, and measurements of haematological and biochemical parameters. Cognitive function tests were introduced at the third survey (2003–2006) and repeated at the fourth survey (2006–2008) and fifth survey (2008–2012). A total of 3,601 participants completed two to three measurements of cognitive performance.

The PREVEND study was approved by the medical ethics committee (METc) of University Medical Center Groningen, Groningen, the Netherlands, and conducted in accordance with the guidelines of the Helsinki declaration. All participants gave written informed consent.

### Cognitive performance

Cognitive performance was measured as a composite score of two tests: the Ruff Figural Fluency Test (RFFT) and the Visual Association Test (VAT). The RFFT is generally seen as a measure of executive function but provides also information regarding planning, divergent thinking and the ability to shift between different cognitive tasks. The RFFT requires the participants to draw as many designs as possible within a set time limit while avoiding repetitions of designs. The main outcome of the RFFT is the total number of unique designs, which range from 0 points (worst score) to 175 points (best score) (13). The RFFT is sensitive to changes in cognitive performance in both young and old persons (13, 14).

The VAT is a brief learning task that is designed to detect memory impairment including anterograde amnesia. The test consists of six drawings of pairs of interacting objects. The participant is asked to name each object and, later, is presented with one object from the pair and asked to name the other object. The lowest (worst) score is 0 points, the highest (best) score is 12 points (15).

To create a composite cognitive score, the raw RFFT and VAT scores at each measurement were standardized to z-scores (based on the mean and standard deviation of each test at the first measurement) and subsequently averaged.

### Treatable vascular risk

Treatable vascular risk was based on the components of the Framingham Risk Score for Cardiovascular Disease (FRS-CD) that are amenable to treatment: diabetes mellitus (yes/no), current smoker status (yes/no), systolic blood pressure (mmHg), total cholesterol (mmol/l), HDL cholesterol (mmol/l) and use of blood pressure lowering drugs (yes/no). The FRS-CD is designed to predict the risk of a new cardiovascular, cerebrovascular or peripheral vascular event within the next ten years. This model was validated for persons aged 30–74 years without vascular history (3).

A higher treatable vascular risk score is associated with a higher risk of a new vascular event: the lowest score is -5 (10-year risk <1%), and the highest score is 21 (10-year risk >30%) (3).

### Measurements of treatable vascular risk components

Data on the treatable vascular risk were obtained in the third survey of the PREVEND study at the same visit at which the first measurement of cognitive performance was done: total cholesterol, HDL-cholesterol and glucose were measured with fasting blood tests. Diabetes mellitus was defined as a fasting glucose ≥7.0 mmol/L (126 mg/dl) or a non-fasting glucose ≥11.0 mmol/L (200 mg/dl) or the use of glucose-lowering drugs. Smoking was defined as current smoker based on self-report. Systolic blood pressure was automatically measured (Dinamap) in a supine position during ten minutes and reported as the average of the two last measurements. Data on actual drug use were obtained from the InterAction DataBase that comprised pharmacy-dispending data from regional community pharmacies (16).

### Covariates

Demographic factors were measured at the first measurement. Data on age, gender and educational level were obtained from a questionnaire. Educational level was divided into four groups: primary school (0 to 8 years of education), lower secondary education (9 to 12 years of education), higher secondary education (13 to 15 years of education), and university (≥16 years of education). Because the effect of vascular risk on cognitive function is possibly modified by APOE ε4 carriership (17), APOE ε4 genotype was included as a covariate. Participants were categorized as APOE ε4 carriers (allele combinations e2/e4 or e3/e4 or e4/e4) or noncarriers (allele e2/e2 or e2/e3 or e3/e3).

### Statistical analysis

Parametric data are presented as mean and standard deviation (SD) and nonparametric data as median and interquartile range (IQR). Differences were tested by independent-samples t test or, if appropriate, Mann-Whitney U test. Differences between paired observations were tested by paired-samples t test or, if appropriate, Wilcoxon signed-rank test. Differences in proportion were tested by Chi-Square test. Trends across measurements were analyzed by ANOVA for parametric data and by Kruskal-Wallis H test for nonparametric data.

The longitudinal association of cognitive performance with the treatable vascular risk was investigated by linear multilevel analysis (linear mixed model analysis). Cognitive performance was the dependent variable. Treatable vascular risk at the first measurement was the independent variable. The analysis included the data of all participants who completed the cognitive tests on at least two measurements. Consecutive measurement (1, 2, or 3) was the lowest level and participant the highest level. Interaction between the treatable vascular risk and consecutive measurement was investigated by entering the product term treatable vascular risk x consecutive measurement into the regression model. Interaction between the treatable vascular risk and APOE ε4 carriership was tested by entering treatable vascular risk x APOE ε4 carriership into the model. Adjustment was made for age, educational level, consecutive measurement and interaction age x consecutive measurement. To study the effect of the separate components of treatable vascular risk, a similar regression model was built with all separate components (Supplement). In all models, the variables cognitive performance, consecutive measurement, age (years) and treatable vascular risk (points) were entered as continuous variables. Educational level and APOE ε4 carriership were entered as categorical variables. The level of statistical significance was set at 0.05. The linear multilevel analyses were performed using MLwiN Version 2.29 (Centre for Multilevel Modelling, University of Bristol, Bristol, UK) (18), the other analyses were performed using IBM SPSS Statistics 22.0 (IBM, Amonk, NY).

### Sensitivity analyses

Various a priori-defined analyses were performed. First, the analyses were limited to persons aged 35–74 years without vascular history, because the FRS-CD was only validated in this age group (3). Second, to investigate the generalizability of our findings, analyses were repeated with two other risk scores based on the treatable components of the Framingham Risk Score for Coronary Heart Disease (FRS-CHD) and the SCORE risk system (5, 6). Third, the analyses were repeated after exclusion of all APOE ε2 carriers (allele combinations ε2/ε2, ε2/ε3 and ε2/ε4) because the APOE ε2 allele appears to reduce the risk of Alzheimer's disease (19). Finally, the analyses were repeated in a subset of the PREVEND cohort, the Groningen Random Sample, which had a similar prevalence of microalbuminuria (8%) and other cardiovascular risk factors as the general population (20).

## Results

### Study population

Overall, 3,601 participants completed the cognitive tests at multiple measurements: 2,431 (68%) participants at three measurements and 1,170 (32%) participants at two measurements. Eighteen (0.5%) participants were excluded because their educational level was not known and three (0.1%) participants because their age was younger than 35 years and their number too small to form a separate age group. Eight (0.2%) persons were excluded because of missing data on treatable vascular risk. Thus, the total study population included 3,572 persons with a mean (SD) age of 54 (11) years, 52% were men and 96% of Western-European descent (Table 1).

**Table 1:** Characteristics of the study population at the first measurement (baseline)

	**All**
n (%)	3,572 (100)
Age (years), mean (SD)	54 (11)
Age groups, n (%)	
35-44 years	824 (23)
45-54 years	1182 (33)
55-64 years	889 (25)
65-74 years	534 (15)
≥75 years	143 (4)
Gender, n (%)	
Men	1867 (52)
Women	1705 (48)
Educational level, n (%)	
Primary school	396 (11)
Secondary lower education	930 (26)
Secondary higher education	968 (27)
University	1278 (36)
Cardiovascular history, n (%)	160 (5)
Cardiac event	133 (3)
Cerebrovascular event	24 (1)
Peripheral vascular event	3 (1)
Cardiovascular risk factors	
Hypertension, n (%)	1222 (34)
Diabetes mellitus, n (%)	197 (6)
Smoker, n (%)	799 (22)
Systolic blood pressure (mmHg), mean (SD)	125 (17)
Total cholesterol (mmol/L), mean (SD)	5.36 (1.04)
HDL-cholesterol (mmol/L), mean (SD)	1.41 (0.38)
Non-HDL-cholesterol (mmol/L), mean (SD)	3.94 (1.02)
Blood pressure lowering drugs, n (%)	827 (23)
APOE ε4 carriership*, n (%)	943 (26)


Abbreviations: HDL, high-density lipoprotein; SD, standard deviation; * APOE ε4 carriership included the allele combinations ε2/ε4, ε3/ε4 and ε4/ε4.

### Longitudinal course of cognitive performance and treatable vascular risk

The mean (SD) total follow-up time was 5.5 (0.7) years. The mean (SD) cognitive performance of the total study population changed per consecutive measurement from 0.00 (0.79) at the first measurement to 0.15 (0.83) at second measurement and to 0.39 (0.82) at third measurement (Ptrend<.001). The change in cognitive performance per consecutive measurement was most clear in the age groups 35 to 44 years, 45 to 54 years, and 55 to 64 years (Table 2). Treatable vascular risk ranged from -5 to +17 points with a mean (SD) of 2 (4) points at the first measurement. Except for the age group 35 to 44 years, treatable vascular risk did not change statistically significantly per consecutive measurement (Table 2).

**Table 2:** Change in cognitive performance* and treatable vascular risk† across measurements per age group

**Age (years)**	**Variable**	**Measurement**			**P trend**
		**First**	**Second**	**Third**	
35-44	Cognitive performance, z-score	0.41 (0.71)	0.67 (0.68)	0.90 (0.64)	<.001
	Treatable vascular risk, points	1 (3)	0 (3)	1 (3)	.02
45-54	Cognitive performance, z-score	0.16 (0.73)	0.30 (0.71)	0.55 (0.65)	<.001
	Treatable vascular risk, points	2 (4)	2 (4)	2 (4)	.09
55-64	Cognitive performance, z-score	-0.17 (0.73)	-0.04 (0.76)	0.15 (0.73)	<.001
	Treatable vascular risk, points	3 (4)	3 (4)	3 (3)	.83
65-74	Cognitive performance, z-score	-0.52 (0.68)	-0.46 (0.73)	-0.40 (0.77)	.07
	Treatable vascular risk, points	4 (3)	4 (3)	4 (3)	.13
≥75	Cognitive performance, z-score	-0.72 (0.69)	-0.75 (0.78)	-0.62 (0.76)	.56
	Treatable vascular risk, points	5 (3)	4 (3)	5 (3)	.42


All values are noted as mean (SD). Abbreviations: SD, standard deviation; * Cognitive performance was measured as a composite score of two tests (z-score): the Ruff Figural Fluency Test (RFFT) and the Visual Association Test (VAT) (13,15); † Treatable vascular risk is based on the components of Framingham Risk Score for Cardiovascular Disease that are amenable to treatment and included diabetes mellitus, current smoker status, total cholesterol, HDL-cholesterol, systolic blood pressure and use of blood pressure lowering drugs (3).

### Longitudinal change in cognitive performance and treatable vascular risk

**Figure 1:**
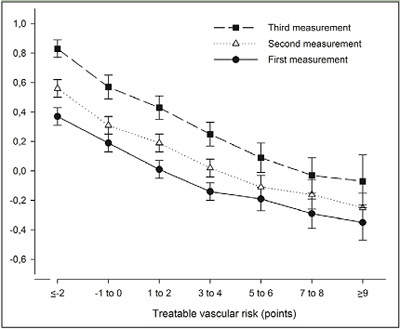
Mean cognitive performance per measurement dependent on the treatable vascular risk at first measurement. Bars represent 95% confidence intervals. Cognitive performance was measured as a composite score of two tests (z-score): the Ruff Figural Fluency Test (RFFT) and the Visual Association Test (VAT) (13,15). Treatable vascular risk is based on the components of Framingham Risk Score for Cardiovascular Disease that are amenable to treatment and included diabetes mellitus, current smoker status, total cholesterol, HDLcholesterol, systolic blood pressure and use of blood pressure lowering drugs (3).

Longitudinal change in cognitive performance was dependent on treatable vascular risk: the change in cognitive performance was negatively associated with treatable vascular risk (Figure 1). The mean change in cognitive performance between the first and third measurement was 0.46 (95%CI, 0.37 to 0.55; P<.001) in persons with the lowest treatable vascular risk whereas it was 0.28 (95%CI, 0.08 to 0.47; P=.006) in persons with the highest treatable vascular risk. The association between cognitive performance and treatable vascular risk was confirmed by multilevel analysis. Adjusted for age, educational level, consecutive measurement and interaction age x consecutive measurement, the multilevel regression model did not only show a statistically significant effect for treatable vascular risk (B-coefficient, -0.011; 95%CI, -0.019 to -0.003; P=.01) but also for the interaction between treatable vascular risk and consecutive measurement (Table 3). The change in cognitive performance between two measurements decreased with 0.004 per one-point increment of treatable vascular risk (B-coefficient, -0.004; 95%CI, -0.008 to 0.000; P=.05). This is comparable to the decrease in change in cognitive performance between two measurements per one-year increment of age (B coefficient, -0.006; 95%CI, -0.008 to -0.004; P<.001) (Table 3).

**Table 3:** Longitudinal association of cognitive performance* on the treatable vascular risk†: multilevel linear analysis

	**Model 1§**			**Model 2║**			**Model 3{**		
	**B**	**95%CI**	**P**	**B**	**95%CI**	**P**	**B**	**95%CI**	**P**
Age (years)	-0.020	-0.022 to -0.018	<.001	-0.018	-0.020 to -0.016	<.001	-0.018	-0.020 to -0.016	<.001
Educational level (vs. primary school)									
Secondary lower education	0.20	0.13 to 0.26	<.001	0.19	0.13 to 0.26	<.001	0.19	0.13 to 0.26	<.001
Secondary higher education	0.40	0.33 to 0.47	<.001	0.38	0.32 to 0.45	<.001	0.38	0.32 to 0.45	<.001
University	0.68	0.61 to 0.75	<.001	0.65	0.58 to 0.72	<.001	0.65	0.58 to 0.72	<.001
Measurement‡	0.50	0.43 to 0.56	<.001	0.50	0.44 to 0.56	<.001	0.50	0.42 to 0.54	<.001
Age x measurement‡	-0.006	-0.006 -0.008 to -0.004	<.001	-0.006	-0.008 to -0.004	<.001	-0.006	-0.008 to -0.004	<.001
Treatable vascular risk†				-0.018	-0.024 to -0.012	<.001	-0.011	-0.019 to -0.003	.01
Treatable vascular risk† x measurement‡							-0.004	-0.008 to 0.000	.05


Abbreviations: B, unstandardized B-coefficient; CI, confidence interval; * Cognitive performance was measured as a composite score of two tests (z-score): the Ruff Figural Fluency Test (RFFT) and the Visual Association Test (VAT) (13,15); † Treatable vascular risk is based on the components of Framingham Risk Score for Cardiovascular Disease that are amenable to treatment and included diabetes mellitus, current smoker status, total cholesterol, HDL-cholesterol, systolic blood pressure and use of blood pressure lowering drugs (3); ‡ Consecutive measurement; § For model 1: -2*log likelihood 16864.08; ║ For model 2: -2*log likelihood 16822.30; { For model 3: -2*log likelihood 16817.67.

### Effect of APOE ε4 carriership

The effect of treatable vascular risk on cognitive performance was not modified by APOE ε4 carriership as there was no statistically significant interaction between treatable vascular risk and APOE ε4 carriership: B-coefficient for treatable vascular risk, -0.009 (95%CI, -0.019 to 0.001; P=.07), for APOE ε4 carriership, 0.003 (95%CI, -0.048 to 0.054; P=.91), and for the interaction treatable vascular risk x APOE ε4 carriership, -0.003 (95%CI, -0.015 to 0.009; P=.62).

### Association with separate components of treatable vascular risk

Cognitive performance was not only associated with treatable vascular risk but also with different components of treatable vascular risk. Adjusted for age, gender, educational level, consecutive measurement and interaction age x consecutive measurement, the full multilevel regression model showed that cognitive performance was negatively associated with diabetes mellitus (B coefficient, -0.11; 95%CI, -0.19 to -0.02; P=.01), current smoker (B-coefficient, -0.08; 95%CI, -0.13 to -0.04; P<.001) and hypertension (B-coefficient, -0.05; 95%CI, -0.10 to 0.00; P=.03), and positively associated with HDL-cholesterol (B-coefficient, 0.08; 95%CI, 0.02 to 0.13; P=.005). However, the longitudinal change in cognitive performance was not dependent on any of the separate components of treatable vascular risk because there was no statistically significant interaction between separate components and consecutive measurement (P=.20) (Supplement).

### Sensitivity analyses

Essentially similar results were found if the analyses of the association of cognitive performance with the treatable vascular risk were limited to persons aged 35 to 74 years without vascular history. If the analyses were repeated with treatable vascular risks based on treatable components of FRS-CHD or SCORE as independent variables, the negatively association between cognitive performance and treatable vascular risk was also found. If the analyses were repeated after exclusion of all APOE ε2 carriers, there was no interaction between treatable vascular risk and APOE ε4 carriership. Finally, the association of cognitive performance with treatable vascular risk was also found in the Groningen Random Sample (Supplement).

## Discussion

In this large community-based study, cognitive performance was negatively associated with treatable vascular risk over a follow-up period of almost six years in persons aged 35 to 82 years old. As reported previously (12), cognitive performance increased across the measurements probably due to the repeated exposure to the cognitive tests. However, the change in cognitive performance was dependent on treatable vascular risk and was lower if treatable vascular risk was higher. In addition, our data suggested that the effect of treatable vascular risk on cognitive performance was comparable to the effect of age.

Our results were comparable to the findings of the National Aging Study (NAS) and the two studies from the National Alzheimer Coordinating Center (NACC) cohort (8, 9, 10). In all studies, poor cognitive performance was associated with high treatable vascular risk independent of age. However, our study differs from these studies in study population, duration of followup, APOE ε4 carriership and type of treatable vascular risk score. Whereas the other studies included specific populations of elderly people, our study showed this association in population that comprised both middleaged and old persons. Furthermore, in the NAS and NACC studies the negative association of cognitive performance with treatable vascular risk was found over an average follow-up period of three years (8, 9, 10). Notably, our study adds that this association persisted after a period of almost six years. Comparable to one NACC study (10), our study also showed that the effect of treatable vascular risk factors on cognitive performance was not changed by APOE ε4 carriership whereas the two other studies did not evaluate the interaction of APOE ε4 carriership with treatable vascular risk (8, 9). Moreover, in our study the treatable vascular risk was based on a general vascular risk score and not on a stroke-specific risk score which was used in the NAS and NACC studies (8, 9, 10). Therefore, vascular risk management programmes based on general vascular risk may not only prevent cardiac, cerebrovascular and peripheral vascular events but possibly also cognitive impairment. In addition, our findings from a study population of middle-aged and old persons support the hypothesis that the start of vascular risk management at late-life may be too late for effective prevention of cognitive impairment and dementia (21, 22).

Interestingly, our data suggested that the effect of treatable vascular risk on cognitive performance was comparable to the effect of age. This is in agreement with the finding of the NAS study that the association between cognitive performance and treatable vascular risk was almost as strong as that between cognitive performance and age (8). As a result, it may be estimated that onepoint decrement of treatable vascular risk per year can probably gain one-year in cognitive age. One-point decrement of treatable vascular risk can be achieved by 10 mmHg reduction in systolic blood pressure or 1 mmol/L reduction in total cholesterol (3). These target values are usually achieved in clinical practice and randomized controlled trials (RCTs) (23, 24). Smoking cessation even results in three-points decrement of treatable vascular risk (3). Several studies did not only show that smoking is a risk factor for cognitive impairment, but also that smoking cessation decreased the risk of cognitive impairment to the risk of persons who have never smoked (25). So, smoking cessation is a good preventive measure and may compensate cognitive decline that occurs in three-years increment of age. Thus, a relevant decrease in vascular risk is probably feasible and is like to gain several years in cognitive age.

Recently, it was observed that over the past decades management of vascular risk factors has coincided with a decline in the prevalence of dementia (26). Our findings supported that vascular risk management may not only result in a lower incidence of cardiovascular disease but possibly also in a lower incidence of cognitive impairment and dementia. However, up till now, various RCTs have found inconsistent results about the effect of treatment of vascular risk factors on cognitive performance (27). Only the Syst-Eur trial suggested a protective effect of antihypertensive treatment on dementia in contrast to other trials (27, 28). Similarly, intensified treatment of diabetes mellitus or cholesterol lowering treatment had no effect on cognitive performance in other large trials such as the ADVANCE study and the PROSPER trial (24, 27, 29). It is generally acknowledged that these negative findings may be explained by the use of a relatively insensitive cognitive test or short follow-up period (27). The FINGER, preDIVA and MAPT trials did not have these shortcomings (30, 31, 32). The FINGER trial showed that a multidomain intervention including treatment of vascular risk factors during two years could improve or maintain cognitive performance. However, the effect of treatment of vascular risk factors on cognitive performance per se was unclear as the multidomain intervention also included cognitive training (30). On the other hand, the preDIVA trial did not show a positive effect of the multidomain vascular intervention on cognitive performance, possibly because there was a similar reduction in cardiovascular risk in the intervention and control group (31). Similarly, the MAPT study did not found a difference in 3-year cognitive decline between control group and the multidomain intervention including physical activity, cognitive training and nutritional advice (32). Moreover, the trials included only old persons who were at risk for cognitive impairment (30, 31, 32). Considering our findings, starting vascular risk management in old age or risk groups may be too late for effective prevention of cognitive impairment and dementia (21, 22).

Some limitations of this study have to be noted. First, our study had an observational design whereas it is generally acknowledged that observational studies may give results that differ from subsequent RCTs on the same questions, and may overestimate treatment effects (33). However, RCTs with duration of four years or even longer seem hardly feasible due to high costs and the ethics of not treating vascular risk factors for a long time in placebo group (34, 35). Therefore, observational studies with a long follow-up period are still essential to gain more insight in the consequence of increased vascular risk in middle-age. Second, cognitive performance was measured with two cognitive tests in this study which may not evaluate all cognitive domains. However, the RFFT measures a wide range of different cognitive abilities such as initiation, planning, divergent reasoning, and the ability to switch between different tasks (13). In addition, because of its wide score range, the RFFT is not limited by a ceiling or floor effect and, thereby, sensitive to subtle changes in cognitive performance in young and old persons (13, 14). Furthermore, the VAT was added as a measure of memory (15). Although both tests are dependent on language and relatively specific measures of frontal network functions, semantic and episodic memory, these two tests combined reflect the cognitive domains commonly affected by Alzheimer's disease and vascular dementia. Finally, in our study the cognitive performance increased across the measurements probably due to repeated exposure to the tests resulting in a practice effect (12). Practice effects appear in most, if not all, cognitive tests which assess various cognitive domains like memory, attention and executive functions (36). Practice effects can be ascribed to different factors such as memory of previous responses and learning test strategies, and could explain that people improve or maintain their cognitive performance despite a cognitive decline (36). However, in our study the association of cognitive performance with treatable vascular risk was adjusted for repeated consecutive measurement by entering the variable consecutive measurement and the interaction age x consecutive measurement in the model.

Despite these limitations, the present study also has several strengths. Our study was based on a large community-based cohort and included a large number of both middle-aged and elderly people whereas other longitudinal studies used selected populations of elderly (8, 9, 10). In addition, by using a (general) vascular risk score we explored the synergistic effects of vascular risk factors instead of focusing on a single risk factor. Risk scores have the advantage that multiple separate risk factors are weighted to generate optimal overall risk estimation for individual patients. Additionally, they yield a single variable that is the optimal estimate for overall cardiovascular burden, which limits the number of variables in small studies or multivariate analyses (3, 4, 5, 6). Moreover, vascular risk scores are particularly valuable to identify increased vascular risk in middleaged people because in this age group vascular risk factors often are only marginally elevated if considered separately but result in a clearly increased vascular risk if considered together (3, 4, 5, 6).

In conclusion, in this large community-based cohort change in cognitive performance was associated with treatable vascular risk in both middle-aged and old people. Our data support the hypothesis that the start of vascular risk management at late-life may be too late for effective prevention of cognitive impairment and dementia.

*Additional Contributions:* The authors thank the PREVEND study group and the supporting staff of PREVEND for their role in the logistics of the study and the acquisition of the data used in this article.

*Author Disclosures:* All authors reported no disclosures.

*Conflict of interest:* There is no conflict of interest.

*Ethical standards:* The PREVEND study was approved by the medical ethics committee (METc) of Universitair Medical Center Groningen, Groningen, the Netherlands, and conducted in accordance with the guidelines of the Helsinki declaration.

## Electronic supplementary material

Treatable vascular risk and cognitive performance in persons aged 35 years or older: Longitudinal study of six years
