# Supplementary material for: Treatable Vascular Risk and Cognitive Performance in Persons Aged 35 Years or Older: Longitudinal Study of Six Years
Source: J Prev Alzheimers Dis. 2018 Dec 14;6(1):42–9. doi: 10.14283/jpad.2018.47 (PMC6515561; doi:10.14283/jpad.2018.47)
Supplement: Supplementary file 1 — Treatable vascular risk and cognitive performance in persons aged 35 years or older: Longitudinal study of six years [file mmc1.docx]

**ONLINE SUPPLEMENTAL**

Treatable vascular risk and cognitive performance in persons aged 35 years or older: Longitudinal study of six years

Marlise E.A. van Eersel^1,^*, MD, PhD; Hanneke Joosten^2^, MD, PhD; Ron T. Gansevoort^3^, MD, PhD; Joris P.J. Slaets^1^, MD, PhD; Gerbrand J. Izaks^1^, MD, PhD.

^1^ University of Groningen, University Medical Center Groningen, University Center for Geriatric Medicine, Groningen, The Netherlands

^2^ Department of Internal Medicine, Maastricht University Medical Center, Maastricht, The Netherlands

^3^ University of Groningen, University Medical Center Groningen, Department of Nephrology, Groningen, The Netherlands

**Methods**

*Statistical Analysis: Association with separate components of treatable vascular risk*

The first step was to analyse the association of cognitive performance with each separate components of treatable vascular risk. Adjustment was made for age, gender, educational level, consecutive measurement number, interaction age x consecutive measurement number and other treatable vascular risk factors. The second step was to analyse the interaction between each separate component of treatable vascular risk and consecutive measurement number by entering the product term of separate component x consecutive measurement number into the regression model. The last step was to analyse the association of cognitive performance with all separate components of treatable vascular risk in a full regression model. Separate components of treatable vascular risk were diabetes mellitus (yes/no), current smoker (yes/no), total cholesterol (mmol/L), HDL-cholesterol (mmol/L), hypertension (yes/no). The level of statistical significance was set at 0.05. The linear multilevel analyses were performed using MLwiN Version 2.29 (Centre for Multilevel Modelling, University of Bristol, Bristol, UK) (1).

*Sensitivity analyses*

Various a priori-defined analyses were performed. First, the analyses were limited to persons aged 35-74 years without vascular history (n=3,286), because the FRS-GCD was only validated in this specific group (2). Second, to investigate the generalizability of our findings, analyses were repeated in the total study population with two other risk scores based on the treatable components of the Framingham Risk Score for Coronary Heart Disease (FRS-CHD) and the SCORE risk system (n=3,574) (3,4). However, these risk scores were also validated in specific groups. So, the analyses were repeated again and limited to persons without vascular history aged 35 to 74 years old for FRS-CHD (n=3,288) and to persons without vascular history aged 40 to 65 years old for SCORE (n=2,493) (3,4). Third, the analyses were repeated after exclusion of all *APOE* ε2 carriers (allele combinations ε2/ε2, ε2/ε3 and ε2/ε4) because the *APOE* ε2 allele appears to reduce the risk of Alzheimer’s disease (5), and the effect of *APOE* ε2/ε4 genotype on cognitive function is unclear (n=2,699). Finally, as a consequence of its design, the participants of the PREVEND study had a somewhat higher prevalence of microalbuminuria than the general population (10% vs. 8%, respectively) (6,7). Because this may influence data analyses, the analyses were repeated in a subset of the PREVEND cohort, the Groningen Random Sample (n=1,454), which had a similar prevalence of microalbuminuria (8%) and other cardiovascular risk factors as the general population (6).

**Results**

*Association with separate components of treatable vascular risk*

Cognitive performance was associated with different components of treatable vascular risk. Diabetes mellitus (*P*=.01), current smoker (*P*<.001), HDL-cholesterol (*P*=.005) and hypertension (*P*=.03) were independently associated with cognitive performance, after adjustment for age, gender, educational level, consecutive measurement number, interaction age x consecutive measurement number and other treatable vascular risk factors (Table 1). There was no statistically significant interaction between each individual component and consecutive measurement number (*P*=.20) (Table 1). Adjusted for age, gender, educational level, consecutive measurement number and interaction age x consecutive measurement number, the full multilevel regression model showed that cognitive performance was negatively associated with diabetes mellitus (*P*=.01), current smoker (*P*<.001) and hypertension (*P*=.03), and positively associated with HDL-cholesterol (*P*=.005) (Table 2).

*Sensitivity analyses*

Essentially similar results were found if the analyses of the association of cognitive performance with the treatable vascular risk were limited to persons aged 35 to 74 years old without vascular history: B‑coefficient for treatable vascular risk, ‑0.011 (95%CI, ‑0.019 to ‑0.003; *P*=.006), and for treatable vascular risk x consecutive measurement number, ‑0.004 (95%CI, ‑0.008 to 0.000; *P*=.05). If the analyses were repeated in total study population with treatable vascular risks based on treatable components of FRS-CHD or SCORE as independent variables, the negatively association between cognitive performance and treatable vascular risk was also found: B-coefficient for FRS-CHD, ‑0.009 (95%CI, ‑0.015 to ‑0.003; *P*=.003), and for SCORE, ‑0.08 (95%CI, ‑0.15 to 0.00; *P*=.04), respectively. The longitudinal change in cognitive performance was also dependent on these treatable vascular risks: B-coefficient for interaction FRS-CHD x consecutive measurement number, ‑0.002 (95%CI, ‑0.004 to 0.000; *P*=.05), and for interaction SCORE x consecutive measurement number, ‑0.04 (95%CI, ‑0.07 to 0.00; *P*=.02), respectively. If the analysis with FRS-CHD was limited to persons aged 35 to 74 years old without vascular history, similar results were found. In the full regression model, the B-coefficient for treatable vascular risk based on FRS-CHD was ‑0.010 (95%CI, ‑0.018 to ‑0.002; *P*=.01), and for treatable vascular risk x consecutive measurement number, ‑0.002 (95%CI, ‑0.004 to 0.000; *P*=.05). If the analysis with the SCORE was limited to persons aged 40 to 65 years old without vascular history, similar results were found even though they were borderline statistically significant. The B-coefficient for treatable vascular risk based on SCORE was ‑0.07 (95%CI, ‑0.16 to 0.02; *P*=.14), and for treatable vascular risk x consecutive measurement number, ‑0.04 (95%CI, ‑0.07 to 0.00; *P*=.04). If the analyses were repeated after exclusion of all *APOE* ε2 carriers, the longitudinal change in cognitive performance remained dependent on treatable vascular risk (*P*=.05). Comparable to the analyses in the full study population, there was no interaction between treatable vascular risk and *APOE* ε4 carriership (*P*=.62). Finally, essentially similar results were found if the analyses were repeated in the Groningen Random Sample. However, the interaction treatable vascular risk x consecutive measurement number was not statistically significant because of the smaller study population.

**References**

1. Rasbash J, Charlton C, Browne WJ, Healy M, Cameron B. MLwiN version 2.1. Centre for multilevel modelling, University of Bristol. 2009:<http://www.bristol.ac.uk/cmm/software/mlwin/>. Accessed on 14 August 2018.
2. D'Agostino RB S, Vasan RS, Pencina MJ, et al. General cardiovascular risk profile for use in primary care: The Framingham heart study. Circulation. 2008;117(6):743-753.
3. Wilson PW, D'Agostino RB, Levy D, Belanger AM, Silbershatz H, Kannel WB. Prediction of coronary heart disease using risk factor categories. Circulation. 1998;97(18):1837-1847.
4. Conroy RM, Pyorala K, Fitzgerald AP, et al. Estimation of ten-year risk of fatal cardiovascular disease in Europe: The SCORE project. Eur Heart J. 2003;24(11):987-1003.
5. Bertram L, McQueen MB, Mullin K, Blacker D, Tanzi RE. Systematic meta-analyses of Alzheimer disease genetic association studies: The AlzGene database. Nat Genet. 2007;39(1):17-23.
6. Lambers Heerspink HJ, Brantsma AH, de Zeeuw D, et al. Albuminuria assessed from first-morning-void urine samples versus 24-hour urine collections as a predictor of cardiovascular morbidity and mortality. Am J Epidemiol. 2008;168(8):897-905.
7. De Jong PE, Hillege HL, Pinto-Sietsma SJ, de Zeeuw D. Screening for microalbuminuria in the general population: a tool to detect subjects a risk for progressive renal failure in an early phase? Nephrol Dial Transplant. 2003;18:10-13.
8. Ruff R. Ruff Figural Fluency Test: Professional manual. Lutz, FL: Psychological Assessment Resources, Inc. 1996.
9. Lindeboom J, Schmand B. Visual association test. Manual. PITS B V Leiden, The Netherlands. 2003.

Table 1

Composite table of separate longitudinal association of cognitive performance* on separate components of treatable vascular risk^†^: multilevel linear analyses

|  | B | 95%CI | *P* | -2*log likelihood |  | B | 95%CI | *P* | -2*log likelihood |
| --- | --- | --- | --- | --- | --- | --- | --- | --- | --- |
| Diabetes (yes vs. no) | ‑0.11 | ‑0.19 to ‑0.02 | .01 | 16775.09 |  | ‑0.13 | ‑0.26 to 0.00 | .05 | 16774.93 |
| Diabetes (yes vs. no) x measurement^‡^ |  |  |  |  |  | 0.01 | ‑0.05 to 0.07 | .71 |  |
|  |  |  |  |  |  |  |  |  |  |
| Current smoker (yes vs. no) | ‑0.08 | ‑0.13 to ‑0.04 | <.001 | 16775.09 |  | ‑0.02 | ‑0.09 to 0.04 | .49 | 16770.24 |
| Current smoker (yes vs. no) x measurement^‡^ |  |  |  |  |  | ‑0.03 | ‑0.06 to 0.00 | .03 |  |
|  |  |  |  |  |  |  |  |  |  |
| Total cholesterol (mmol/L) | ‑0.01 | ‑0.03 to 0.01 | .22 | 16775.09 |  | 0.00 | ‑0.03 to 0.03 | .89 | 16774.39 |
| Total cholesterol (mmol/L) x measurement^‡^ |  |  |  |  |  | ‑0.01 | ‑0.02 to 0.01 | .40 |  |
|  |  |  |  |  |  |  |  |  |  |
| HDL-cholesterol (mmol/L) | 0.08 | 0.02 to 0.13 | .005 | 16775.09 |  | 0.08 | 0.00 to 0.16 | .05 | 16775.09 |
| HDL-cholesterol (mmol/L) x measurement^‡^ |  |  |  |  |  | 0.00 | ‑0.03 to 0.03 | .95 |  |
|  |  |  |  |  |  |  |  |  |  |
| Hypertension^§^ (yes vs. no) | ‑0.05 | ‑0.10 to 0.00 | .03 | 16775.09 |  | ‑0.02 | ‑0.09 to 0.05 | .63 | 16773.45 |
| Hypertension^§^ x measurement^‡^ |  |  |  |  |  | ‑0.02 | ‑0.05 to 0.01 | .20 |  |

All models are adjusted for age, gender, educational level, consecutive measurement number, interaction age x consecutive measurement number and other treatable vascular risk factors. Abbreviations: HDL, high-density lipoprotein; B, unstandardized B-coefficient; CI, confidence interval.

* Cognitive performance was measured as a composite score of two tests (z-score): the Ruff Figural Fluency Test (RFFT) and the Visual Association Test (VAT) (8,9).

^†^ Treatable vascular risk is based on the components of Framingham Risk Score for Cardiovascular Disease that are amenable to treatment and included diabetes mellitus, current smoker status, total cholesterol, HDL-cholesterol, systolic blood pressure and use of blood pressure lowering (2).

^‡^ Consecutive measurement.

^§^ Hypertension is combination of two separate components of the Framingham Risk Score for Cardiovascular Disease and defined as systolic blood pressure above 140mmHg and/or use of blood pressure lowering medication.

Table 2

Longitudinal association of cognitive performance* with individual components of treatable vascular risk^†^: multilevel linear analysis

|  | Model 1^\|\|^ | | |
| --- | --- | --- | --- |
|  | B | 95%CI | *P* |
| Age (years) | ‑0.018 | ‑0.020 to ‑0.016 | <.001 |
| Gender (women vs. men) | 0.11 | 0.07 to 0.15 | <.001 |
| Educational level (vs. primary school) |  |  |  |
| Secondary lower education | 0.19 | 0.13 to 0.26 | <.001 |
| Secondary higher education | 0.40 | 0.33 to 0.47 | <.001 |
| University | 0.66 | 0.59 to 0.72 | <.001 |
| Measurement^‡^ | 0.50 | 0.44 to 0.55 | <.001 |
| Age x measurement^‡^ | ‑0.006 | ‑0.008 to ‑0.004 | <.001 |
| Diabetes mellitus (yes vs. no) | ‑0.11 | ‑0.19 to ‑0.02 | .01 |
| Current smoker (yes vs. no) | ‑0.08 | ‑0.13 to ‑0.04 | <.001 |
| Total cholesterol (mmol/L) | ‑0.01 | ‑0.03 to 0.01 | .22 |
| HDL-cholesterol (mmol/L) | 0.08 | 0.02 to 0.13 | .005 |
| Hypertension^§^ (yes vs. no) | ‑0.05 | ‑0.10 to 0.00 | .03 |

Abbreviations: HDL, high-density lipoprotein; B, unstandardized B-coefficient; CI, confidence interval.

* Cognitive performance was measured as a composite score of two tests (z-score): the Ruff Figural Fluency Test (RFFT) and the Visual Association Test (VAT) (8,9).

^†^ Treatable vascular risk is based on the components of Framingham Risk Score for Cardiovascular Disease that are amenable to treatment and included diabetes mellitus, current smoker status, total cholesterol, HDL-cholesterol, systolic blood pressure and use of blood pressure lowering drugs (2).

$\ddagger$ Consecutive measurement.

^§^ Hypertension is combination of two separate components of the Framingham Risk Score for Cardiovascular Disease and defined as systolic blood pressure above 140mmHg and/or use of blood pressure lowering medication.

^||^ For model 1: -2*log likelihood 16775.09.
